# Fractionated radiotherapy is the main stimulus for the induction of cell death and of Hsp70 release of p53 mutated glioblastoma cell lines

**DOI:** 10.1186/1748-717X-9-89

**Published:** 2014-03-30

**Authors:** Yvonne Rubner, Carolin Muth, Annedore Strnad, Anja Derer, Renate Sieber, Rolf Buslei, Benjamin Frey, Rainer Fietkau, Udo S Gaipl

**Affiliations:** 1 Department of Radiation Oncology, University Hospital Erlangen, Friedrich-Alexander-Universität Erlangen-Nürnberg, Erlangen, Germany; 2 Department of Neuropathology, University Hospital Erlangen, Friedrich-Alexander-Universität Erlangen-Nürnberg, Erlangen, Germany

**Keywords:** Glioblastoma multiforme, Fractionated radiotherapy, Temozolomide, Valproic acid, Clonogenic potential, G2 cell cycle arrest, Apoptosis, Necrosis, Hsp70, Immunogenic cell death

## Abstract

**Background:**

Glioblastoma multiforme (GBM) is the most common primary brain tumor in adults. Despite a multimodal therapy consisting of resection followed by fractionated radiotherapy (RT) combined with the chemotherapeutic agent (CT) temozolomide (TMZ), its recurrence is almost inevitable. Since the immune system is capable of eliminating small tumor masses, a therapy should also aim to stimulate anti-tumor immune responses by induction of immunogenic cell death forms. The histone deacetylase inhibitor valproic acid (VPA) might foster this.

**Methods:**

Reflecting therapy standards, we applied in our *in vitro* model fractionated RT with a single dose of 2Gy and clinically relevant concentrations of CT. Not only the impact of RT and/or CT with TMZ and/or VPA on the clonogenic potential and cell cycle of the glioblastoma cell lines T98G, U251MG, and U87MG was analyzed, but also the resulting cell death forms and release of danger signals such as heat-shock protein70 (Hsp70) and high-mobility group protein B1 (HMGB1).

**Results:**

The clonogenic assays revealed that T98G and U251MG, having mutated tumor suppressor protein p53, are more resistant to RT and CT than U87MG with wild type (WT) p53. In all glioblastoma cells lines, fractionated RT induced a G2 cell cycle arrest, but only in the case of U87MG, TMZ and/or VPA alone resulted in this cell cycle block. Further, fractionated RT significantly increased the number of apoptotic and necrotic tumor cells in all three cell lines. However, only in U87MG, the treatment with TMZ and/or VPA alone, or in combination with fractionated RT, induced significantly more cell death compared to untreated or irradiated controls. While necrotic glioblastoma cells were present after VPA, TMZ especially led to significantly increased amounts of U87MG cells in the radiosensitive G2 cell cycle phase. While CT did not impact on the release of Hsp70, fractionated RT resulted in significantly increased extracellular concentrations of Hsp70 in p53 mutated and WT glioblastoma cells.

**Conclusions:**

Our results indicate that fractionated RT is the main stimulus for induction of glioblastoma cell death forms with immunogenic potential. The generated tumor cell microenvironment might be beneficial to include immune therapies for GBM in the future.

## Background

Glioblastoma multiforme (GBM) is the most common and malignant primary brain tumor in adults. It is characterized by its aggressive, diffuse infiltrative and invasive growth. The extensive cellular and molecular heterogeneity of GBM may contribute to poor prognosis of the patients [1]. Because of the infiltrative growth of the tumor a complete resection is difficult and radiation (RT) mostly combined with chemotherapy (CT) follow as adjuvant treatments. A conventional RT scheme consists of a total dose of 60Gy, given at 5 consecutive days weekly in 2Gy fractions over 6 weeks. The most widely used chemotherapeutics in GBM treatment are alkylating agents, including the well tolerated imidazoletetrazinone derivative temozolomide (TMZ), since the lipophilic properties allow to pass the blood brain barrier. Stupp et al. reported about an improved median survival from 12.1 to 14.6 months and an increased 5-years survival from 1.9% to 9.8% of patients with GBM treated with concomitant and adjuvant TMZ and RT compared to RT alone [2].

Levels of oral administered TMZ in the brain or the cerebrospinal fluid reach about 30-40% [3] of the plasma concentration, which ranges between 27 μM and 50 μM [4]. Its cytotoxicity is due to the methylation of the O^6^ position of guanine. This causes mispairing with thymine during the next replication cycle [5]. However, the presence of the DNA repair protein O^6^-methylguanine methyltransferase (MGMT) in a large number of gliomas confers to resistance of alkylating agents [6]. Therefore the promotor methylation of the MGMT gene, observed for GBM and various other tumor types, results in an improved outcome for patients, through decreased expression of the dealkylating protein [7]. As already demonstrated by *in vitro* experiments, TMZ is capable of inducing cell cycle arrest in the G2/M phase [8], senescence [9], apoptosis [10], or autophagy [11] in glioma cells. Data about the release of danger signals and the loss of the tumor cell membrane integrity, characteristic for primary and secondary necrotic cells, are still lacking.

Epileptic seizures are common in 30 to 50% of GBM patients [12]. Patients receiving valproic acid (VPA) as anticonvulsant during TMZ based radiotherapy have a better outcome than patients treated with other antiepileptic drugs (AED) or not receiving any AED [13]. VPA can be administered orally and also crosses the blood-brain barrier [14]. Levels in the brain reach about 7 to 28% of the serum/plasma concentration, which ranges between 20-100 μg/ml in epilepsy patients [15]. Furthermore, VPA is an effective histone deacetylase (HDAC) inhibitor [16]. It induces growth arrest, apoptosis, senescence, and autophagy in medullablastoma and glioma cells [17,18]. A sensitization by VPA of human glioma cells to TMZ and irradiation was just reported recently [19].

The tumor suppressor gene *p53* plays a major role in the regulation of cellular stress responses. In non-malignant cells the p53 protein has a short half-life time and is expressed at low levels. However, its protein level increases after exposure to stress stimuli like ionizing radiation, genotoxic DNA-damaging agents or hypoxia, thereby modulating cell cycle, DNA repair, apoptosis, senescence, cellular differentiation, metabolism, angiogenesis and immune response. Nevertheless, the function of p53 is often altered or impaired due to mutations after neoplastic transformation. Mutations in *p53* have been seen in 25-30% of primary GBM [20,21]. The incidence of p53 mutations in glioma cell lines is similar to the primary tumor [22]. Several established human GBM cell lines with wild type (WT, e.g. in U87MG) or mutant p53 (e.g. in T98G, U251MG, U138MG, A-172) exist for studying the impact of p53 in cancer treatment [23].

The contribution of the immune system in eliminating small tumor masses, recurrent tumors or metastases has become increasingly evident [24,25]. Chemotherapeutic agents and γ-irradiation induce DNA damage, which leads to cell cycle arrest and proliferation stop. Irreparable damages result in the induction of senescence or distinct forms of cell death [26,27]. The two main cell death forms are apoptosis and necrosis. In contrast to necrotic cells, apoptotic cells are usually non-inflammatory or even anti-inflammatory, because of their maintenance of the plasma membrane integrity and swift clearance by macrophages. However, some chemotherapeutic agents, like anthracyclines and oxaliplatin, as well as ionizing irradiation are capable of inducing immunogenic forms of apoptotic cell death [28]. Because of the loss of membrane integrity, necrotic cell death leads, besides a supply of tumor-associated antigens (TAA), to the release of damage-associated molecular pattern molecules (DAMP), which trigger inflammation and immune activation [29,30]. Formerly only known as non-programmed form of cell death, regulated necrosis has become evident during the last years. The so called necroptosis is negatively regulated by caspase-8 and is dependent on the kinase activity of RIPK3 and RIPK1 [31]. The latter is inhibitable by necrostatin-1, while caspase-dependent apoptosis is inhibitable by the pan-caspase inhibitor zVAD-fmk [32,33]. Secondary necrotic cells result from apoptotic cells that were not properly cleared and have lost their membrane integrity at any time point during apoptosis execution [34,35]. Primary non-programmed necrosis is characterized by early plasma membrane rupture and dilatation of cytoplasmic organelles [36]. The early plasma membrane rupture can be induced by mechanical stress or take place in very early phases after apoptosis induction when no DNA degradation has already taken place [37]. We here define primary necrotic cells as cells that have lost their membrane integrity but still have full DNA content. Secondary necrotic cells are defined as late apoptotic cells that have already degraded their DNA and lost the membrane integrity. RT- and CT-induced glioblastoma cell death was inhibitable by zVAD-fmk, but not by necrostatin-1 (not shown). This suggests that mainly secondary necrosis and no RIPK1-dependent necroptosis was induced in the examined glioblastoma cell lines.

Released danger signals by necrotic cells in general, such as the heat shock protein 70 (Hsp70) and the DNA-binding protein high-mobility group B1 (HMGB1) activate dendritic cells (DC) and foster the efficient processing and (cross) presentation of antigens, respectively. DC are professional antigen presenting cells (APC) and crucial for the priming of tumor-specific naïve T cells [38]. For the induction of an efficient antitumor immune response the activation of both cytotoxic CD8^+^ T cells and CD4^+^ T helper cells is required [39]. Intracellular antigens are usually presented on MHC class I to CD8^+^ T cells and extracellular antigens such as TAA are presented on MHC class II to CD4 +T cells. However, HMGB1 and Hsp70 enable DC to also cross-present TAA in a MHC class I dependent manner [40]. In that way, not only T helper cells, but also cytotoxic T cells are primed.

Little is known about tumor cell death forms that are induced by the combination of RT and CT in GBM. Especially the knowledge about combined treatments which could change the immunosuppressive tumor microenvironment to an immune stimulating one is rare. Further, most of the *in vitro* studies that determine the response of tumor cells to chemotherapeutics alone or in combination with RT use much higher doses of CT and RT compared to clinical routine applications. Preclinical models reproducing clinical conditions are needed for radiobiological studies [41]. Commonly, the clonogenic (or colony forming) assay is used as read out to determine the reproductive viability of cells after single and multimodal treatments. The surviving fraction is expressed by the linear-quadratic model which predicts the sensitivity and repair capacity of cells to radiation and/or CT. This dose response relationship model is still the biological basis for most of the RT schemes in the clinics. Nevertheless, is has several drawbacks, including that the cells are seeded in different concentrations, depending on the treatment, the low plating efficiency, the disregarded cell-to-cell communication, and clump artifacts. Further, the colony forming assay does not allow any assumption about cell death induction and forms of tumor cell death including their immunogenicity [42,43].

Because of the above mentioned, our purpose was to examine cell death forms of p53 WT and p53 mutated glioblastoma cell lines with an *in vitro* system that resembles closer to clinical protocols in GBM treatment. We here focused on apoptosis and necrosis of the three human glioblastoma cell lines U87MG (p53 WT), U251MG (p53 mutated), and T98G (p53 mutated) induced by conventional fractionated RT (5x2Gy, weekly dose) in combination with clinically achievable levels of TMZ. We further examined whether VPA influences/increases the radiosensitivity of glioma cells. To get hints about the immunogenic potential of glioblastoma cells, besides the analysis of apoptosis and necrosis by AnnexinA5-FITC/PI staining, the release of the immune activating danger signals HMGB1 and HSP70 was assessed.

## Methods

### Cell culture and reagents

The human glioblastoma tumor cell lines U87MG and T98G were obtained from the American Type Culture Collection (ATCC; Manassas, USA), and U251MG from Cell Line Service (CLS; Eppelheim, Germany). The cell lines were tested to be free of mycoplasma contamination before performing the experiments. Cells were maintained in Dulbecco’s Modified Eagle’s Medium (DMEM; PAN-Biotech GmbH, Aidenbach, Germany) supplemented with 10% fetal bovine serum (FBS; Biochrom AG, Berlin, Germany), 1% sodium pyruvate, 2 mM glutamine, 100U/ml penicillin and 100 μg/ml streptomycin (termed D10 medium; Invitrogen, Darmstadt, Germany). Cells were grown in cell culture flasks (Cellstar; Greiner BioOne, Nürtlingen, Germany) at 37°C in humidified air with 5% CO2. TMZ and VPA were purchased from Sigma-Aldrich (Munich, Germany). TMZ was dissolved at a stock concentration of 100 mM in dimethylsulfoxide (DMSO) and stored at -20°C. Chemotherapeutics were diluted in D10 immediately before treatment of cells.

### Treatment with chemotherapeutic agents and X-ray

The glioblastoma cells were seeded at a density of 2.5 × 10^5^ cells in 75 cm^2^ flasks. The chemotherapeutics were added 24 h (0.5 mM VPA) or 40 h (20 μM TMZ) later. Two hours after TMZ treatment, cells were irradiated with 2Gy (representing a daily dose in GBM therapy). Irradiation was repeated on four consecutive days to achieve a clinically relevant total weekly dose of 10Gy. Forty-eight hours after the last radiation, supernatants for ELISA (enzyme-linked immunosorbent assay) analyses were collected, and cells were harvested for cell death detection and cell cycle analysis by flow cytometry. Irradiation was performed with an X-ray generator (120 kV, 22.7 mA, variable time; GE Inspection Technologies, Hürth, Germany).

### Clonogenic assay

The effect of TMZ and VPA or a combination of both on the radiosensitivity of the three glioblastoma cell lines was assessed with clonogenic assays. Cells were plated in triplicates in 60-mm dishes (Nunc Thermo Fisher, Waltham, USA) at concentrations estimated to yield 40-150 colonies/dish. The chemotherapeutics were added 24 h (0.5 mM VPA) or 40 h (20 μM TMZ) after seeding. Two hours after TMZ treatment, cells were irradiated with 1, 2, 4, 6, 8 or 10Gy. After incubation for ~2 weeks, cells were fixed with methylene blue (Sigma-Aldrich, Munich, Germany) for 30 min. Colonies with >50 cells were scored.

### Cell death detection

Induction of cell death in glioblastoma cells and cell death forms by the respective treatments were examined using AnnexinA5 (AnxA5)-FITC/Propidium Iodide (PI) staining. The cell suspensions (1 × 10^6^ cells/ml) were incubated for 30 min at 4°C in the dark with 0.5 μg/ml AnxA5-FITC (Geneart, Regensburg, Germany; FluoroTaq FITC conjugation Kit, Sigma-Aldrich, Munich, Germany) and 1 μg/ml PI (Sigma-Aldrich, Munich, Germany) in Ringer’s Solution (Baxter S.A., Lessines, B). Samples were analyzed by flow cytometry (EPICS XL MCL™, Beckman Coulter, Brea, USA) and its associated Kaluza 1.1 Software®. A minimum of 10.000 events/sample were acquired. Viable cells show neither binding of AnxA5 nor staining with PI. Early apoptotic cells are positive for AnxA5 binding and PI negative, whereas late apoptotic cells (referred as secondary necrotic ones, AnxA5+/PI+) and necrotic cells (referred as primary necrotic ones, AnxA5+/PI++) were positive for both, AnnexinA5 and PI binding. Inhibitors for necroptosis and apoptosis were used in selected experiments to determine whether the detected necrosis by AnxA5-FITC/PI staining is necroptosis or secondary necrosis. The necroptosis inhibitor necrostatin-1 (nec-1; Sigma-Aldrich, Munich, Germany) was used in the concentration of 10 μM and the pan-caspase inhibitor carbobenzoxy-valyl-alanyl-aspartyl-[Omethyl]-fluoromethyl-ketone (zVAD-fmk Bachem, Weil am Rhein, Germany) in the concentration of 50 μM.

### Cell cycle analysis

Cell cycle phases and the cells with sub-G1 DNA content were analyzed by PI staining in the presence of detergent followed by analysis with flow-cytometry as described by Riccardi and Nicoletti [44]. In brief, cells (5 × 10^5^) were fixed at least for 20 min in 70% ethanol at -20°C. After permeabilization of cells with TritonX-100 containing solution, cells were incubated for 30 min with 200 μg/ml RNase A (Roche, Mannheim, Germany) and 5 μg/ml PI at room temperature. DNA content was determined with Kaluza 1.1 Software® (Beckman Coulter, Krefeld, Germany) after excluding doublets and clumps by gating on the FL3 versus FL3(AUX).

### ELISA for Hsp70 and HMGB1 in cell culture supernatants

Cell culture supernatants were centrifuged to remove remaining cells and stored at -80°C until the determination of extracellular HMGB1 or Hsp70 concentrations by ELISA technique. The Hsp70 ELISA kit was purchased by R&D Systems (DuoSet®, Wiesbaden-Nordenstadt, Germany) and that of HMGB1 by Shino-Test Corporation (Kanagawa, Japan). ELISA were performed according to the manufacturer’s instructions.

### Statistical analysis

Data were expressed as the mean ± standard deviation (SD) and analyzed for statistical significance using Students t-test (t-tailed) or non-parametric Mann-Whitney U-test (GraphPad Prism®, La Jolla, USA). A p value <0.05 was considered to be significant.

## Results

### The extent of radiosensitization by the chemotherapeutic agents TMZ and/or VPA differs in glioblastoma cell lines

In general, GBM is a radio- and chemo-resistant tumor. However, in consistence with its great heterogeneity, a wide range of radiosensitivity has been demonstrated with *in vitro* clonogenic and *in vivo* assays based on xenogeneic mouse models [45]. Williams et al. analyzed the clonogenic potential of 39 tumor cell lines and found the three human glioblastoma cell lines T98G, U251MG, and U87MG as the most resistant cell lines in a dose range 2-10Gy [46,47]. The MGMT status of the glioblastoma cells is involved in responsiveness to TMZ. We detected a high steady state mRNA and MGMT protein expression levels in U251MG and T98G, but not in U87MG glioblastoma cells (not shown).

**Figure 1:**
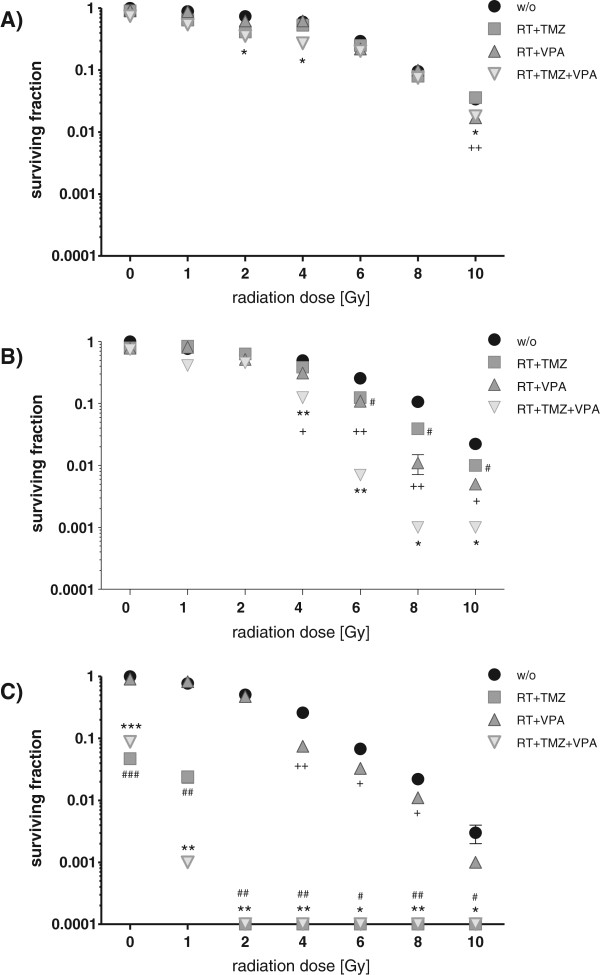
**Colony formation of glioblastoma cells after treatment with RT and/or CT with TMZ and/or VPA.** T98G **(A)**, U251MG **(B)**, or U87MG **(C)** tumor cells were treated with TMZ (20 μM) and/or VPA (0.5 mM) alone, or in combination with RT. The dose-dependent clonogenic potential was determined after 2 weeks of incubation. The colony number (100%) of untreated cells was set as 1, and the reduction of the colony number due to treatment was calculated accordingly. Data from one experiment representative of at least two independent experiments is shown (n = 3). Mean values (±SD) are displayed. Gy, Gray; RT, radiotherapy; CT, chemotherapy; w/o, untreated control; TMZ, temozolomide; VPA, valproic acid; # P < 0.05, ## P < 0.01, ### P < 0.001 (TMZ ± RT against w/o ± RT), *P < 0.05, **P < 0.01, ***P < 0.001 (TMZ/VPA ± RT against w/o ± RT), + P < 0.05, ++ P < 0.01, +++ P < 0.001 (VPA ± RT against w/o ± RT).

Our data show that T98G and U251MG, which express MGMT and contain mutant p53, are more resistant to radiation than U87MG with no MGMT expression and p53 WT (Figure 1). Approximately 29% of T98G, 25% of U251MG and 7% of U87MG survived after a radiation dose of 6Gy. At a dosage above 6Gy, the cell line U251MG showed also an enhanced sensitivity to radiation compared to T98G (Figure 1A, B).

A promising approach for the treatment of GBM patients are radiosensitizers. Therefore, the radiosensitizing effect of the alkylating chemotherapeutic agent TMZ and the anti-epileptic drug and histone deacetylase inhibitor VPA alone or in combinations were tested. In T98G tumor cells, neither TMZ nor VPA alone or the combination did reduce the colony formation. Further, only VPA or the combination of VPA with TMZ exerted slight radiosensitizing effects (Figure 1A). In contrast, colony formation of the cell line U251MG was suppressed when TMZ or VPA were combined alone with RT (≥4Gy). A further significant reduction of the colony numbers was achieved when TMZ plus VPA were combined together with RT (Figure 1B). Unlike T98G and U251MG glioblastoma cells, the p53 WT and MGMT negative cell line U87MG was highly sensitive to TMZ. TMZ reduced the capability to form colonies by around 96% compared to untreated cells (Figure 1C). The combination with RT (≥ 2Gy) resulted in the complete loss of colony formation. VPA also sensitized U87MG to radiation (≥ 4Gy). Since TMZ alone was already a very effective radiosensitizer, the combination of TMZ and VPA could not further potentiate the growth inhibitory effect exerted by TMZ.

### Fractionated RT induces apoptosis and necrosis in glioblastoma cells

As outlined above, TMZ in combination with VPA sensitizes the human glioblastoma cell lines T98G, U251MG and U87MG to radiation, monitored by a significant reduction of colony formation. Since this assay gives no information about cell death induction and immunological relevant cell death forms [24,43], we analyzed the latter by AnxA5-FITC/PI assay. Although during the last years several data about tumor cell death induction by radiation and/or chemotherapeutic agents were published, the focus was mainly set on high single doses of X-rays [48] or high concentrations of CT [3,10,19,49]. To get hints how clinically relevant treatment schemes influence cell death of glioblastoma cells, we analyzed the cell death forms of the glioblastoma cell lines after fractionated irradiation with 5x2Gy in combination with clinically relevant concentration of TMZ and/or VPA in 2D culture systems.

**Figure 2:**
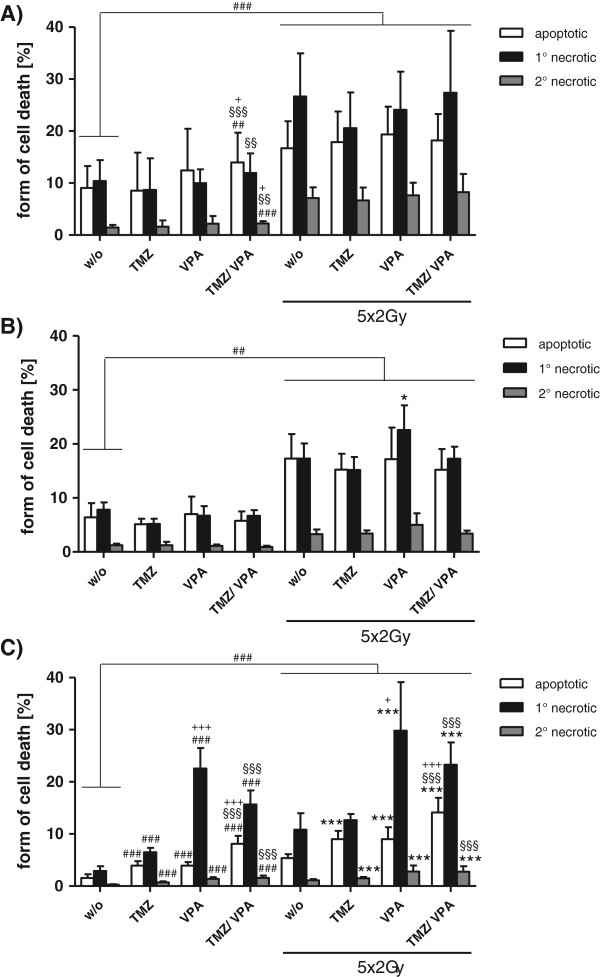
**Forms of glioblastoma cell death after fractionated RT and/or CT with TMZ and/or VPA.** T98G **(A)**, U251MG **(B)**, or U87MG **(C)** tumor cells were treated with TMZ (20 μM) and/or VPA (0.5 mM) alone, or in combination with 5 daily radiation doses of 2Gy. Forty-eight hours after the last treatment, cells were stained with AnxA5-FITC/PI and cell death was analyzed by flow cytometry. The percentages of apoptotic, primary, or secondary necrotic tumor cells are displayed. Pooled results of four independent experiments are shown, each performed at least in triplicates. Mean values (±SD) are displayed. Gy, Gray; RT, radiotherapy; CT, chemotherapy; w/o, untreated control; TMZ, temozolomide; VPA, valproic acid; 1°, primary; 2°, secondary; ## P < 0.01, ### P < 0.001 (against w/o), *P < 0.05, ***P < 0.001 (against RT), §§ P < 0.01, §§§ P < 0.001 (TMZ ± RT against VPA + TMZ ± RT), + P < 0.05, +++ P < 0.001 (VPA ± RT against VPA + TMZ ± RT).

Figure 2A and B display cell death forms of T98G and U251MG glioblastoma cells two days after the last treatment with fractionated RT, TMZ and/or VPA alone or their combinations. In both cell lines, fractionated RT resulted in a significantly increased number of both apoptotic and necrotic cells. A radiosensitizing effect regarding apoptosis and necrosis induction by TMZ, VPA or TMZ plus VPA was not observed (Figure 2A and B). This was also true when higher concentrations of TMZ (200 μM) or a daily dose of TMZ (20 μM)/VPA (0.5 mM) were administered (data not shown). A slight, but significant increase of apoptosis and necrosis was seen in non-irradiated T98G glioblastoma cells when they were treated with a combination of TMZ and VPA (Figure 2A).

### In p53 WT and MGMT negative U87MG glioblastoma cells, CT and fractionated RT alone and combinatory treatments induce predominantly necrotic tumor cell death

Treatment of U87MG cells with TMZ and/or VPA without irradiation resulted in significantly increased amounts of apoptotic and necrotic tumor cells. Necrosis dominated over apoptosis and VPA was a stronger inducer of necrotic cell death compared to TMZ (Figure 2C). Fractionated RT alone again significantly increased glioblastoma tumor cell death. Combinations with CT further significantly increased apoptosis and, in the presence of VPA, also necrosis of U87MG glioblastoma cells (Figure 2C). As observed for T98G and U251MG cells, fractionated RT induced a mixture of apoptotic and necrotic cell death. However, in U87MG cells with WT p53, combinations with VPA predominantly induced necrotic tumor cell death and VPA further potentiated the effects of TMZ or TMZ + RT (Figure 2C). Of note is that the observed necrosis is not RIPK1-dependent necroptosis, since glioblastoma cell death induced by RT and/or CT was inhibitable by zVAD-fmk, but not by necrostatin-1 (not shown).

### Fractionated RT induces a G2-cell cycle arrest in glioblastoma cells

Ionizing radiation and TMZ are known to induce an arrest of tumor cells in the G2 phase of the cell cycle. In this phase the cells are highly susceptible to further irradiation.

**Figure 3:**
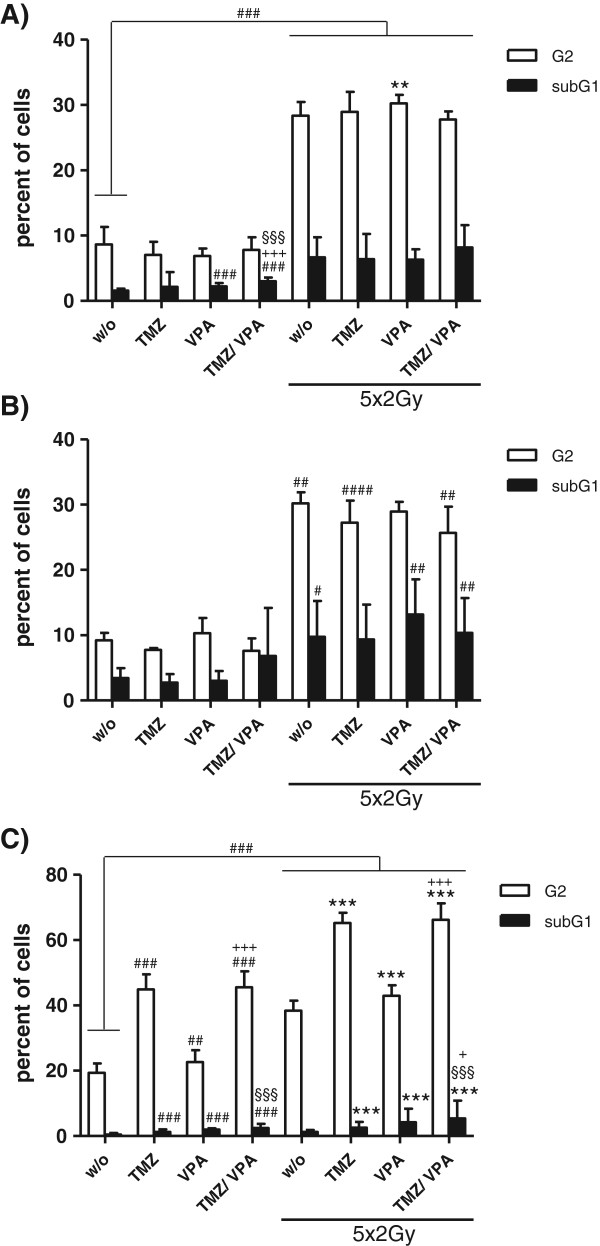
**Glioblastoma cells in the G2 cell cycle phase and with subG1-DNA-content after fractionated RT and/or CT.** T98G **(A)**, U251MG **(B)**, or U87MG **(C)** tumor cells were treated with TMZ (20 μM) and/or VPA (0.5 mM) alone, or in combination with 5 daily radiation doses of 2Gy. Forty-eight hours after the last treatment, cells were harvested. For determination of the DNA content, cells was stained with PI and analyzed by flow cytometry. The percentage of cells in the G2-phase of the cell cycle and that of the subG1-population with degraded DNA are displayed. Pooled results of four independent experiments are shown, each performed at least in triplicates. Mean values (±SD) are displayed. Gy, Gray; RT, radiotherapy; CT, chemotherapy; w/o, untreated control; TMZ, temozolomide; VPA, valproic acid; # P < 0.05, ## P < 0.01, ### P < 0.001 (against w/o), **P < 0.01, ***P < 0.001 (against RT), §§§ P < 0.001 (TMZ ± RT against VPA + TMZ ± RT), + P < 0.05, +++ P < 0.001 (VPA ± RT against VPA + TMZ ± RT).

Around one third of T98G or U251MG cells arrested in the G2 phase of the cell cycle as early as 4 h after exposure to fractionated RT. The G2-arrest sustained for at least 48 hours after the last fractionation dose (Figure 3A and B). The importance of applying fractionated schemes resembling the clinical situation for preclinical evaluations is indicated by the fact that irradiation of T98G with a single dose of 10Gy did not induce a G2 cell cycle block when monitored from 4 up to 72 h after the irradiation (data not shown). In contrast, in U251MG cells a significant G2-cell cycle block occurred after a single irradiation with 10Gy. However, it occurred later (after 24 h) and declined faster (data not shown).

### Temozolomide induces a G2 cell cycle arrest only in U87MG, but not in T98G and U251MG cells

TMZ did not induce an accumulation of the p53 mutant glioblastoma cell lines T98G and U251MG in the G2-phase of the cell cycle (Figure 3A and B). In contrast, in p53 WT U87MG cells, not only fractionated RT induced a significant G2-block, but also a comparable level was achieved by treatment with TMZ alone or in combination with VPA (Figure 3C). Combinations of fractionated RT with TMZ led to a massive arrest of the tumor cells in the G2-phase of the cell cycle; around 65% two days after the last irradiation. Treatment of U87MG with TMZ was further associated with the appearance of hyperploid cells with a DNA content greater than 4n (data not shown). In U87MG cells, VPA did also slightly, but significantly increase the percentage of the tumor cells in the G2-phase of the cell cycle.

TMZ, VPA, TMZ plus VPA and combinations with fractionated RT further increased the amount of the subG1-DNA content of the cells, indicating that late apoptosis/secondary necrosis is induced in U87MG cells (Figures 2C and 3C).

### Increased release of the danger signal Hsp70 after fractionated RT of glioblastoma cell lines

**Figure 4:**
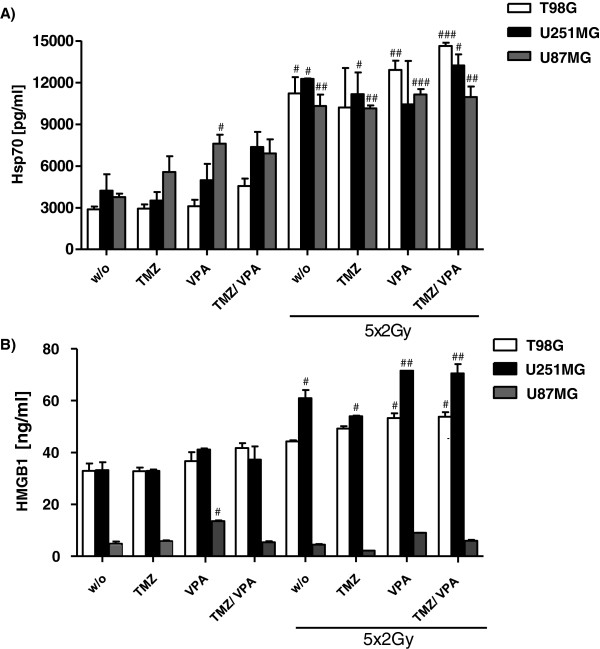
**Hsp70 and HMGB1 release of the glioblastoma cell lines T98G, U251MG, and U87MG after RT and/or CT.** The tumor cells were treated with TMZ (20 μM) and/or VPA (0.5 mM) alone, or in combination with 5 daily radiation doses of 2Gy. Forty-eight hours after the last treatment, supernatants were analyzed for the amount of extracellular Hsp70 **(A)** or HMGB1 **(B)** by ELISA. The data show one representative experiment of at least two independent experiments, each performed in duplicates. Mean values (±SD) are displayed. Gy, Gray; RT, radiotherapy; CT, chemotherapy; w/o, untreated control; TMZ, temozolomide; VPA, valproic acid, HMGB1: DNA-binding protein high-mobility group B1; Hsp70, heat shock protein 70; # P < 0.05, ## P < 0.01, ### P < 0.001 (against w/o).

Nowadays it is well accepted that the immune system contributes to the elimination of tumor cells. However, glioblastoma tumors evolved multiple mechanisms to evade immunological surveillance. Some chemotherapeutics and/or ionizing radiation are known to stimulate an anti-tumor immune response in several cancers by the induction of immunogenic cell death forms (summarised in [50,51]). Since the immunogenic potential of tumor cells can be determined by their release of danger signals, we analyzed the concentration of HMGB1 and of Hsp70 in supernatants of glioblastoma cell cultures treated with fractionated RT and/or CT. Five daily doses of 2Gy significantly increased the amount of extracellular Hsp70 in all three investigated glioblastoma cell lines (Figure 4A). Combined treatment with CT did not further increase the secretion of Hsp70. In contrast, the amount of extracellular HMGB1 was not significantly altered after exposure of the glioblastoma cell line U87MG to fractionated RT and/or CT. However, a slight, but significant increased amount of extracellular HMGB1 was observed in T98G and U251MG tumor cells after fractionated RT, especially in combinations with VPA (Figure 4B).

## Discussion

Radio-resistance [52] and innate or acquired resistance to CT [53-55] are among the reasons for poor control and treatment responses of GBM. Consequently, an urgent need exists for innovative treatments or treatment combinations to improve the outcome of patients with GBM. Recent clinical data have shown that the median survival can be improved after an additional treatment of patients with VPA [13,56] and immune therapeutic approaches are promising [57].

The tumor suppressor gene p53 is involved in cellular radiosensitivity and mutated in more than 50% of gliomas [20,58]. As expected, the p53 mutant gliomblastoma cell lines T98G and U251MG showed an increased clonogenic survival after RT compared to the p53 WT cell line U87MG (Figure 1A-C). Only in the p53 WT and MGMT negative situation a cytotoxic (Figure 2C) and highly radiosensitizing effect (Figure 1C) of TMZ was observed. After combined treatment of RT and TMZ, colony formation in U251MG was only slightly impaired and in T98G completely unaffected (Figure 1A, B).

Besides the tumor suppressor p53, the MGMT status of cells is involved in responsiveness to TMZ [59]. Yoshino et al. found no MGMT mRNA and protein expression in U87MG and U251MG, but in T98G. The IC50 values of MGMT negative cell lines U87MG and U251MG were 23.0 μM and 22.5 μM, respectively, and 441.6 μM for MGMT positive T98G cells [60]. Therefore, p53 and the MGMT status of cells might influence the susceptibility to TMZ. However, contrary to Yoshino et al., we (data not shown) and others [3,61] obtained much higher IC50 values for TMZ in U87MG, U251MG (<200 μM), and T98G (>500 μM). TMZ concentrations that are reached in patients were found to be only sufficient to completely eliminate glioblastoma stem cells *in vitro* from MGMT negative but not from MGMT positive tumors [62]. Hermisson et al. described that U251MG cells have no significant MGMT activity and are therefore rather sensitive to TMZ [63]. However, we found high steady state mRNA and MGMT protein expression levels not only in T98G, but also U251MG glioblastoma cells. The latter were rather resistant to TMZ with regard to the clonogenicity (Figure 1). Therefore, we assume that MGMT expression and its accompanied TMZ resistance can be acquired during cell culture due to epigenetic alterations and the focus should be more set on p53 mutation status for experiments dealing with radiosensitizing effects on glioblastoma cell lines. Even in the clinic the MGMT promoter methylation status used as a prognostic and predictive marker for GBM should be handled with care [64].

From cell culture experiments, it has been reported that VPA sensitizes human glioma cells to γ-radiation and TMZ [19,61]. Data from colorectal cancer cells suggest a p53 dependency of radiosensitization by VPA [65]. Our results show that, depending of radiation dose, VPA is capable of enhancing the radiosensitivity of both p53 mutant and p53 WT glioblastoma cell lines as analyzed by the reduction of their clonogenic potential (Figure 1). Further, this HDAC inhibitor potentiated the effect of TMZ in MGMT expressing and p53 mutated T98G and U251MG. HDAC inhibitors are known as activators of methylated genes [66]. VPA alone had no cytotoxic effect in our experiments when administered at a concentration of 0.5 mM. The IC50 values of VPA determined by us (data not shown) and others exceeded 5 mM [61]. However, because of its radiosensitizing properties (Figure 1) in addition to its antiepileptic effect, VPA should be considered to be applied in combination with standard therapy of GBM. VPA further induced a slightly increased release of HMGB1 in T98G and U251MG glioblastoma cells when combined with fractionated RT (Figure 4B).

If DNA is damaged by radiation or cytotoxic agents, cells stop their cell cycle and start to repair. Non reparable damage results in the initiation of cell death. Fractionated RT induced a G2-arrest in p53 WT and mutated glioblastoma cell lines (Figure 3A-C). The sensitivity to TMZ was associated with an intact p53 and inactive MGMT status, because only in the p53 WT and MGMT negative cell line U87MG a G2-block occurred with an even higher intensity then seen after fractionated RT (Figure 3C). A combination of both (RT + TMZ) resulted in around 20% more cells in the G2-phase than single TMZ or RT. The experiments further showed that fractionated RT induced a mixture of apoptosis and necrosis in all three glioblastoma cell lines (Figure 2A-C). But only in U87MG cells TMZ, VPA and TMZ + VPA alone or their combinations with fractionated RT induced significantly more cell death compared to non-treated or RT-treated controls.

For long-lasting success of multimodal cancer therapies, an induction of systemic anti-tumor immunity, besides the local tumor control by stopping the proliferation of malignant tumor cells, is more and more considered to be of importance [50]. Anti-tumor immunity strongly depends on the induction of immunogenic forms of tumor cell death [67,68]. Only very few preclinical data exist on how fractionated irradiation used in clinical routine impacts on cell death forms of tumor cells. Therefore, we treated GBM cell lines with 5 daily radiation doses of 2Gy alone or in combination with a single administration of TMZ (20 μM) and/or VPA (0.5 mM). Fractionated RT was capable of inducing necrotic glioblastoma cells (Figure 2), as it was also the case for TMZ in p53 WT cells (Figure 2C). Others demonstrated that TMZ administration results in apoptosis [10,69,70] or autophagy [3] in glioma cells. TMZ-induced apoptosis is stimulated by p53 and requires an un-methylated MGMT promotor. We also found a slight increase of apoptosis after TMZ treatment, but only in U87MG cells. Roos et al. showed that apoptosis signals through Fas/CD95/Apo-1 receptor in p53 WT and through the mitochondrial pathway p53 mutated glioma cells, respectively [69]. In western blot analyses we did not observe significant changes in protein levels of pro- and anti-apoptotic proteins involved in the mitochondrial pathway, including Bax, caspase 3 and 7, PUMA, Bcl-2 or XIAP. However, glioblastoma cell death induced by RT and/or CT was inhibitable by the pan-caspase inhibitor zVAD-fmk, but not by necrostatin-1 (not shown). We therefore conclude that the detected necrosis by AnxA5-FITC/PI staining results from apoptotic cells that have lost their membrane integrity during the execution phase of apoptosis. Further, TMZ induced p21 in U87MG cells (data not shown).

We describe for the first time that necrotic glioblastoma cells occur after TMZ treatment at clinically relevant concentrations (20 μM) (Figure 3C). Since fractionated RT alone or in combination with TMZ are capable of inducing bigger amounts of dying cells, the phagocyte system might be overcharged, resulting in necrotic glioblastoma cells not only *in vitro*, but also *in vivo*[24,35]. Jakubowicz-Gil and colleagues also observed a shift of apoptotic to necrotic cell death in T98G after prolonged TMZ incubation (≥48 h) and higher concentrations (>100 μM) [70]. The prolonged incubation time (≥5d) with the low TMZ (20 μM) concentrations in our experimental settings might be the reason for the early appearance of necrotic glioblastoma cells in p53 WT U87MG, but not in p53 mutated T98G or U251MG. Further, apoptosis and necrosis induction in U251MG cells was observed after combined treatment with heat (42°C) and the Hsp90 inhibitor NVP-HSP990 [71].

**Figure 5:**
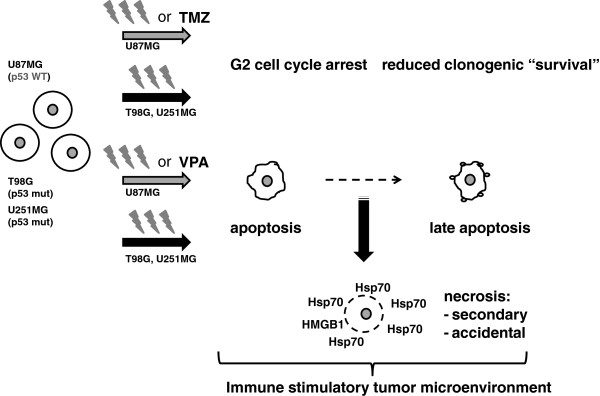
**Hypothesis how especially fractionated RT may induce immunogenic glioblastoma cells.** In the p53 mutated and MGMT positive glioblastoma cell lines T98G and U251MG, fractionated RT is the main stimulus for G2 cell cycle arrest, reduction of clonogenic “survival”, and necrosis concomitant with the release of the danger signal Hsp70. Especially combination with VPA results further in slight increased concentrations of extracellular amounts of the danger signal HMGB1. In p53 WT and MGMT negative U87MG cells, besides fractionated RT, TMZ is one main stimulus for G2 cell cycle arrest and reduction of clonogenic “survival”, while VPA fosters necrotic cell death. The presence of necrotic cells concomitant with Hsp70 after fractionated RT of all examined glioblastoma cells creates a microenvironment with immune stimulatory potential. TMZ, temozolomide; VPA, valproic acid, HMGB1: DNA-binding protein high-mobility group B1; Hsp70, heat shock protein 70; mut: mutated; WT: wild type.

The presence of necrosis and the resulting release of danger signals is accomplished by an immunogenic tumor microenvironment [24,72], which is required for an ameliorated therapeutic outcome in anticancer therapy. Immune stimulatory necrosis was also predominantly observed after treatment with VPA (0.5 mM) and its combinations (TMZ, RT, TMZ + RT) in U87MG (Figure 2C). Further the parallel treatment of U87MG with VPA, TMZ, RT and/or RT + TMZ increased both necrotic and apoptotic cell death compared to controls. Figure 5 summarizes how an immune stimulatory microenvironment may be induced by fractionated RT +/- TMZ/VPA in p53 WT and p53 mutated glioblastoma tumor cells.

Van Nifterik et al. published that VPA does not antagonize the cytotoxic effect of TMZ and is therefore not contraindicated during radiochemotherapy of patients with GBM [19]. It was important for us to use clinical relevant concentrations of CT in our *in vitro* settings. The reached VPA levels in the brain differ between publications. Go et al. described serum/plasma concentrations of 20-100 μg/ml in epileptic patients, whereby brain levels only reach about 7-28% of the total levels [15]. Li et al. state that VPA concentrations in the cerebrospinal fluid are nearly the same as the free concentration in plasma by a half-life time of 9 to 20 hours [17]. In our study, we decided to add VPA to the culture before TMZ and the first RT treatment, because it has been reported that VPA improves the accessibility of DNA to alkylating agents and RT by loosen up the chromatin structure through histone acetylation [73]. Van Nifterik et al. only found a radiosensitizing effect of VPA when added before irradiation [19]. In contrast, Camphausen et al. and Chinnaiyan et al. showed an enhanced radiation response when VPA was added before or after RT. Importantly, VPA can sensitize glioma cells for up to 24 hours after radiation [74]. As described above, our study shows that VPA predominantly results in necrotic tumor cells in U87MG, but does not affect p53 mutated T98G or U251MG (Figure 2). The results of Fu and colleagues indicate that VPA induces autophagy [18], while others report about an apoptotic tumor cell death after VPA [75]. Chen et al. further demonstrated that the synergistic effect of VPA and TMZ for the induction of apoptosis is independent of an intact p53 and suggest a clinical potential for a combined therapy in p53 mutated gliomas. However, our data reveal a more complex mechanism. Although VPA seems to be a promising sensitizer for TMZ and fractionated RT and cell death inductor in p53 WT and MGMT negative cells (U87MG), no significant effects were seen in p53 mutated and MGMT positive T98G. Therefore, it might be useful to test more sensitive HDAC inhibitors in combination with TMZ and RT for p53 mutated glioblastoma cells in the future.

In addition to analyzing the influence of fractionated RT alone or in combination with TMZ and/or VPA on the cell cycle progression (Figure 3) and cell death forms (Figure 2) of glioblastoma cells, we were further interested in the release of the danger signals HMGB1 and Hsp70 after the treatments. While the amount of extracellular HMGB1 was only slightly increased in T98G and U251MG cells especially after combination of RT with VPA, fractionated RT in general resulted in increased extracellular concentrations of Hsp70 in all examined glioblastoma cell lines (Figure 4). Extracellularly present Hsp70 is known to be an immune modulator, which fosters the stimulation and activation of immune cells, including DC and natural killer cells [76,77]. The treatment with five daily doses of 2Gy increased the amount of extracellular Hsp70 significantly in T98G, U251MG, and U87MG. However, TMZ and VPA alone or a combination did not significantly increase extracellular Hsp70 levels, except after exposure of U87MG cells to VPA only (Figure 4). Radiation has been found to modulate cytosolic and surface Hsp70 levels of specifically tumor cells [78]. However, the release could not be induced by a single radiation dose, as shown by Schildkopf et al. for human colorectal tumor cell lines. Only the combination with hyperthermia (HT) increased the amount of extracellular Hsp70 as well as necrotic cell death [79,80]. Therefore, we assume that predominantly repeated exposure to RT leads to early appearance of necrotic glioblastoma cells and to an increased release of Hsp70. Both could be relevant for the induction of anti-tumor immune responses [81]. In future experiments we will analyze how the Hsp70 containing supernatants of glioblastoma cells modulate maturation and activation of DC as well as the consecutive T cell priming. It has become evident that DC-based immune therapies are promising for GBM. Pilot studies and phase I and II trials have been performed indicating that vaccination with DC improves immune functions and results in longer survival of patients with malignant gliomas. The authors conclude that DC-vaccination in combination with RT and TMZ in patients with GBM is safe, feasible, and capable of inducing tumor-specific immune responses [57,82-86]. In our first analyses of biopsies of GBM patients we detected a higher expression level of Hsp70 in relapses compared to the primary tumor (unpublished data). This might indicate that especially in the relapse situation DC-based immune therapies will work well.

## Conclusions

Our *in vitro* data suggest that fractionated RT is the main stimulus for cell death induction and Hsp70 release of especially p53 mutated and MGMT negative glioblastoma cells. Since Hsp70 is capable of activating DC, future preclinical and clinical work has to identify whether high extracellular concentrations of Hsp70 after fractionated RT generates a tumor microenvironment of GBM that favors additional immunotherapy.

## Competing interests

The authors declare that they have no competing interests.

## Authors’ contributions

YR carried out most of the practical work and drafted the manuscript. CM carried out the Hsp70 and HMGB1 ELISA and contributed to analysis and interpretation of the data. AS contributed to analysis and interpretation of the data. AD contributed to the interpretation of the data and drafting of the final manuscript. RS carried out the clonogenic assays. RB contributed to conception and design of the work and to the interpretation of the data. BF contributed to analysis and interpretation of data. RF contributed to the design of the work. USG conceived the study, participated in its design and coordination and finally drafted the manuscript. All authors read and approved the final manuscript.
